# Effect of Tennis Expertise on Motion-in-Depth Perception at Different Speeds: An Event-Related Potential Study

**DOI:** 10.3390/brainsci12091160

**Published:** 2022-08-30

**Authors:** Congyi Wang, Aohan Yan, Wei Deng, Changzhu Qi

**Affiliations:** School of Health Sciences, Wuhan Sports University, Wuhan 430079, China

**Keywords:** tennis expertise, motion-in-depth perception, speed, expert–novice paradigm, ERP

## Abstract

Tennis experts need to extract effective visual information from a sphere in high-speed motion, in which motion-in-depth perception plays an important role. The purpose of the current study was to investigate the impact of sphere speed and tennis expertise on motion-in-depth perception by using the expert–novice task paradigm along with event-related potential (ERP) technology. The study also explored differences in behavior and electroencephalogram (EEG) characteristics between tennis experts and novices. Results show that faster sphere movement led to shorter response times and a lower accuracy rate. The P1 component in the occipital–temporal region showed that the expert group activated earlier and were stronger when the sphere was far away. The latent period of P2 in the occipital region was significantly shorter in the expert group in comparison to the novice group. Faster speed led to the induction of increased P300 volatility and a significant increase in latency. The findings of the current study show that the speed of the sphere movement affects the invocation and allocation of cognitive resources in the process of motion-in-depth perception, irrespective of whether the athletes were experts or novices. There is a special effect in the process of motion-in-depth perception for experts, mainly because attention resources are invested earlier in experts rather than novices.

## 1. Introduction

Previous studies have reported high execution accuracy and excellent athletic performance in fast and complex sports tasks among elite athletes [1]. Athletes require good cognitive processing abilities for excellent performance, especially in fast ball sports. John McEnroe, a famous tennis player, once said, “Things have slowed down, the ball seems to be a lot bigger, and you feel like you have more time”. Where ordinary tennis players lose sight of each other in complex scenes on the court, elite tennis players can quickly identify, accurately track, and intercept tennis balls. An extensive review of several studies in psychology and sports science led Gray to opine that the perception of an athlete depends on interactions between physical characteristics of the object in the sports environment and the ability of the athlete [2]. Motor expertise promotes superior perceptual abilities, which can have better predictive capabilities and better adaptive responses [3,4,5]. The work of Smeeton and Bennett [6] demonstrates that perceptually-skilled participants are characterized by an ability to extract more information about biological motion from the arm. Another review concluded that the expert’s advantage in fast ball sports comes from extracting information from an opponent’s body [7]. The review also shows that a third well-documented skill is the ability of expert athletes to use the visual system in a different, and potentially more effective, manner when scanning and extracting information on display [7]. Based on this question, Müller proposed and verified a preliminary two-stage model of expert visual anticipation for striking sports [8,9]. The model outlines how advanced information from the opponent’s kinematics can be used by experts to guide the lower body portion of the hitting skill, and how ball flight information can be used to guide the interception phase of an implement.

To this end, the effective use of visual information plays a potential role in consistently demonstrating proficient motor performance [10,11]. Information processing theory asserts that when the amount of externally inputted information increases, the cognitive load of an individual increases rapidly, which requires individuals to engage more of their cognitive resources [12]. Experts have more specialized cognitive resources and their cognitive processing efficiency is higher than that of ordinary individuals, which highlights the cognitive advantage of experts [13]. A previous study suggested that the influence of motor expertise on unconscious information can even be transferred to the general cognitive domain [14]. Several previous studies have proposed that elite athletes show high execution accuracy and excellent athletic performance in fast and complex sports tasks [15]. High-level ball players can quickly extract effective visual information and execute fast and accurate decisions [16,17].

Tennis is a net-intercepting sport characterized by fierce competition. Tennis players need to accurately master both the timing of changes in the game’s fast pace and the ever-changing time and space. Their basic visual ability strongly correlates with their ability of sports vision. Compared to non-athletes, athletes can also extensively and effectively use complex visual sporting abilities [18]. Studies have shown that elite athletes and novices use different methods to extract visual information, and that differences in movement expectations between elite and non-elite athletes may explain the better visual perception of elite athletes when compared with novices [19]. The work of Huys shows that expert tennis players use kinematic information from different body regions than novices do in order to anticipate tennis shots [20]. In addition, Williams found that expert karate athletes look at different body regions of an opponent to anticipate attacks than novices do [21].

Moreover, previous studies reported that the athletic performance of athletes is highly correlated to their visual ability, which requires athletes to have good dynamic vision, visual tracking, and perception of motion-in-depth [22,23]. The findings of a selected study explain how the visual system of an expert athlete can better adapt to high-speed processes of the ball [24]. Therefore, tennis players should have good physical qualities and excellent visual perception in addition to their information processing ability. Tennis players need to accurately perceive information about the movement of the ball. Perception of motion-in-depth plays an important role in the cognitive process of ball players. Motion-in-depth perception is essential to guiding and modifying the hitting action in interception-dominated sports.

People need depth perception to determine the distance between an oncoming object and themselves. A key aspect of depth perception for humans is the ability to detect motion-in-depth, i.e., when an object is dynamically approaching or moving away [25]. Previous research has found that object size, velocity, movement patterns [26], the observer’s motor expertise [13], timing of observation, visual field, and distracting objects influence motion-in-depth perception [27]. Extant research has mainly focused on the temporal and spatial characteristics of brain activity [13,28,29]. The EEG components of motion-in-depth perception have been shown to include P1, N2, and P300 in the sensory–motor area [26], as well as P1, N1, P2, and SW in the occipital region [5,13]. Wang and Yao [30] found that N220, P140, and P300 may be ERP components closely related to motion-in-depth perception. Meanwhile, the work of Wei and Qi [13] demonstrates that P2 might be a potential marker for evaluating the ability of the perception of motion-in-depth.

Most previous studies have used planar 2D paradigms to measure motion depth perception, including planar optical flow [29], random dot paradigms [31], and checkerboard zooms [28,32]. These paradigms differ considerably from the actual perception of visual experience. Wang designed an experimental paradigm closer to the real human visual experience in order to solve this problem [26].

Accurate lying about how much the threat of the object to oneself is and the time required to accurately judge it are key issues in the life of an observer [26]. This requires consideration of the perception of motion-in-depth. Athletes show excellent information processing and integration abilities after information input in a high-speed sports sphere, which reflects accurate judgment of the human perception of motion-in-depth. Therefore, the effect of the sports expertise of tennis players on the low-level cognitive process of motion-in-depth perception, and whether its advantages can be used in general simple scenes, should be explored. In addition, the effect of the speed factor on these effects is still being explored. Studies on the process of motion-in-depth are also important parts of research on visual motion perception.

The current study explored characteristics of the motion-in-depth perception of tennis players at different speeds. The study hypothesized that behavioral responses at different speeds of perception of motion-in-depth are different and that event-related potentials are different. We hypothesized that when the ball speed is high, tennis experts process information faster and more accurately in their perception of motion-in-depth. Subsequently, experts will show higher ERP amplitude and shorter latency at P1, N2, P2, and P300.

## 2. Methods

### 2.1. Participants

Two groups of volunteers, one comprising 14 skilled tennis players (7 females) and the other of 14 age-matched novices (7 females), both participated in experiments. No significant difference was observed between the expert (23 ± 1.82) and the novice (24 ± 1.40) groups of participants regarding age and sex. Expert participants satisfied the following inclusion criteria: (1) Skill level: qualified above Second Grade; (2) Professional experience: more than five years’ experience in professional training; (3) Recent practice frequency: playing tennis more than three times per week and above 2 h each time. The novices had no practical experience with any sports. All participants had normal or corrected-to-normal vision and were right-handed. They signed informed consent forms and received a 30 RMB remuneration after a 30-min experiment. This study was approved by the Institutional Research Ethics Committee of Wuhan Sports University.

### 2.2. Materials

The stimulating materials for this study were 3 types of 1000 ms videos (1440 × 900) made by Blender software (Blender, The Netherlands), which were simulated at 30 frames per second to simulate a smooth and continuous motion process with a refresh rate of 60 Hz. The videos were displayed on the 65 cm LCD of the subject. The two types of videos were the target stimulation and the non-target stimulation.

The target stimulus was a 3D sphere with a black-and-white checkerboard pattern corresponding to the two movement patterns of “approaching” and “away”. It contains eight movement speeds (800 cm/s, 1000 cm/s, 1200 cm/s, 1400 cm/s, 1600 cm/s, 1800 cm/s, 2000 cm/s, 2200 cm/s) per movement pattern. In each pattern, a 3D checkerboard sphere appeared in the center of the screen and was smoothly rolled. Participants were asked to identify the motion direction of the sphere as quickly as they could and to press the corresponding key according to the direction.

The non-target stimulus was a circular plane with a black-and-white checkerboard pattern and required no reaction. There are two types of circular plane movements: expansion and contraction.

Behavioral data were recorded the by E-Prime2.0 (E-Prime2.0, Sharpsburg, PA, USA,). EEG activity was recorded using recording software and a 64-channel cap produced by Brain Products (Brain Products, Zeppelinstraße, Gilching, Germany). Non-target stimulus’ data is not included in final analysis.

### 2.3. Design

The present study employed a two (direction: approaching vs. away) by eight (speed: 800 cm/s, 1000 cm/s, 1200 cm/s, 1400 cm/s, 1600 cm/s, 1800 cm/s, 2000 cm/s, 2200 cm/s) by two (group: expert vs. novice) mixed experimental design. Direction and speed were within the subject, and the group was between the subject factors. Dependent variables were behavioral responses (performance accuracy and reaction time) and EEG data (average amplitude and latency). The collected data were analyzed using SPSS 18.0 (SPSS 18.0, Chicago, USA) for the repeated measures analysis of variance (ANOVA).

The experiment comprised a total of 640 trials, which were divided into 4 blocks on average. Each block contained 16 kinds of stimulus materials, and a key balance setting was performed between the blocks. Each trial presented a type of deep motion stimulation. Each stimulation material was randomly presented a total of 40 times. Participants were allowed to autonomously control the rest time between each block.

### 2.4. Procedure

Study participants were asked to get sufficient sleep and were in good mental states. Before the experiment, the participants were required to fill in the basic situation form and sign the informed consent form after the participants specified the tasks. Participants were seated about 65 cm in front of the computer monitor. After putting on the electrode cap, the participants began to practice, which ensured that they understood the experimental process, and the practice data was not recorded in the results.

**Figure 1:**
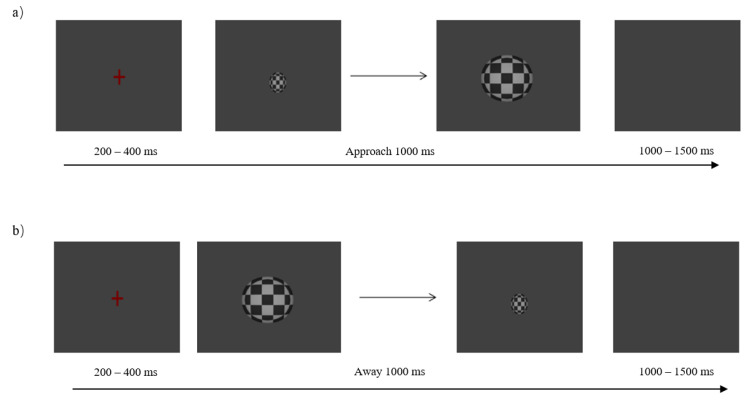
Schematic diagram of the experimental procedure of the deep motion approaching (**a**) and away (**b**) processes. When the sphere appeared, participants were required to press “J” or “F” as soon as possible.

**Figure 2:**
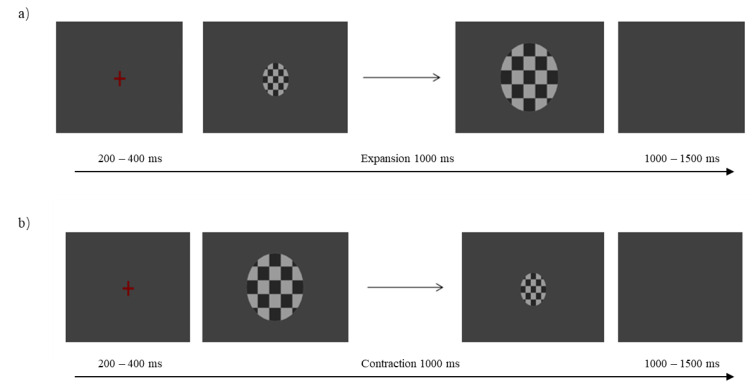
Schematic diagram of the experimental procedure of the expansion (**a**) and contraction (**b**) processes. There was no need for participants to react.

During each trial of the formal experiment, a red “+” fixation point (200~400 ms) was first displayed on the screen, followed by a deep motion stimulation video (1000 ms) (Figure 1 and Figure 2). Participants were required to distinguish between target and non-target stimuli within 1000 ms of presenting the stimulus. For a non-target stimulus, the subject was not expected to not press the button. The target stimulus requires the subject to make a corresponding judgment based on the direction of the depth movement of the sphere. Instructions to participants were as follows: if the sphere approaches the subject, press the “J (F)” key on the keyboard with your usual finger, and press the “F (J)” key if the sphere is far away from the subject. After pressing the key to the next trial, there was a buffer blank screen (1000~1500 ms).

### 2.5. Data Analysis

E-prime 2.0 was used to record the behavioral data of the participants, including response time and accuracy rates of responses. The collected behavioral data were analyzed using SPSS 18.0 for repeated measures analysis of variance (ANOVA).

Electroencephalogram (EEG) data were recorded based on the International 10–20 System with BrainAmp amplifier (Brain Products, Zeppelinstraße, Gilching, Germany)and Ag/AgCl electrodes produced by Brain Products Company (Brain Products, Zeppelinstraße, Gilching, Germany). During recording, the sample rate was 1000 Hz and the frequency range of the amplifier was 0.01–100 Hz. The impedance between the electrodes and the scalp was kept below 5 KΩ. The bandpass filter was set as 0.1–50 Hz with a slope of 24 db/Oct and a notch at 50 Hz. EOG correction was based on 10 Independent Component Analysis (ICA), which detected blinking and then eliminated the EOG component in the blink interval. All trials containing EEG and EOG segments with an amplitude beyond ±75µV were rejected as artifacts. Participants’ data were excluded if their ERP in any condition was less than 30 times on average.

## 3. Results

### 3.1. Behavioral Data

**Figure 3:**
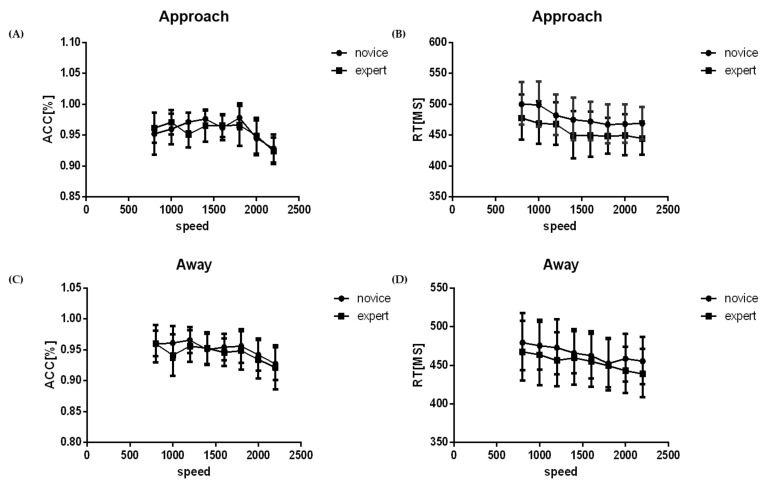
Reaction time and accuracy rate at different speeds. Average line chart of accuracy (**A**) and reaction time (**B**) of experts and novices when the sphere approached at different speeds. Average line chart of accuracy (**C**) and reaction time (**D**) of expert and novice when the sphere receded at different speeds.

Results of repeated measures ANOVA analysis of behavioral data including response time and accuracy management are presented in Figure 3.

The findings on the accuracy rate showed that the main effects of depth direction, F (1, 26) = 4.75, *p* = 0.039, and speed, F (7, 182) = 9.45, *p* < 0.001, were significant. The accuracy rate of “approach” (0.96 ± 0.01) was higher compared with that of “away” (0.94 ± 0.01). Results on response time indicated that the main effects of depth direction, F (1, 26) = 6.2, *p* = 0.019, and speed, F (7, 182) = 25.45, *p* < 0.001, were significant. Response time of “approach” (471.28 ± 10.61) was higher compared to that of “away” (463.17 ± 10.97). Further analysis showed a significant difference between the “approach” and “away” in the novice group, whereas the difference in the expert group was not significant. Analysis of the eight levels of speed factor indicated that when the speed was increased, the accuracy rate of the participants decreased and the response time was shorter (Figure 3).

### 3.2. ERP Results

**Figure 4:**
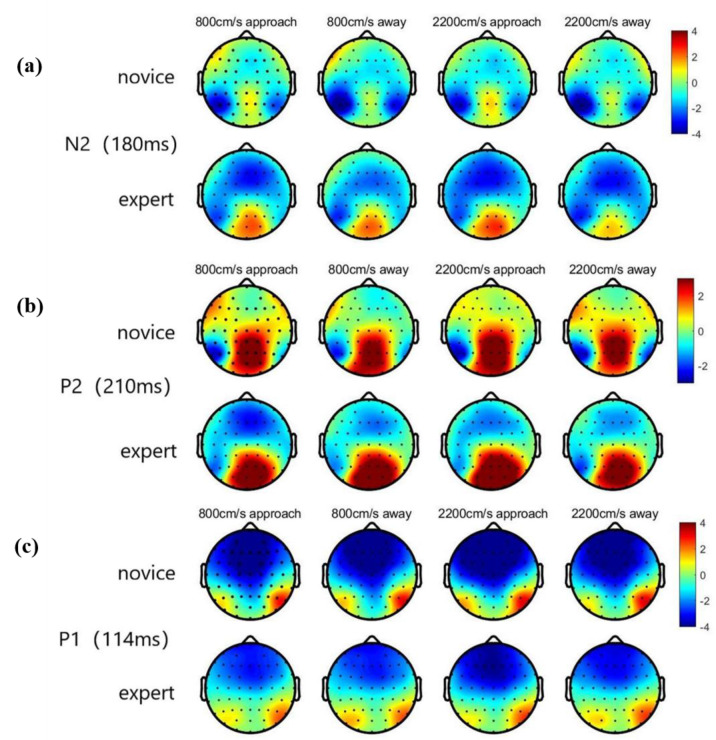
Instantaneous topographic maps of N2 (180 ms) (**a**), P2 (210 ms) (**b**), and P1 (114 ms) (**c**) under four experimental conditions. The topographic maps indicated the difference between novice and expert.

**Figure 5:**
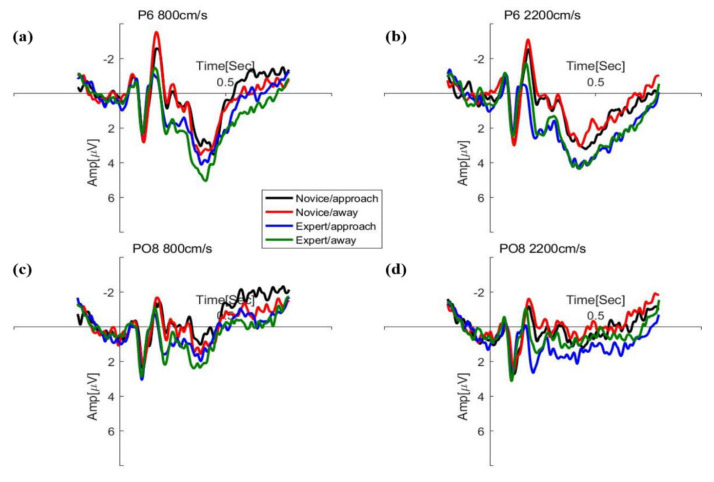
The grand–average waveform for expert and novice at speeds of 800 cm/s (**a**,**c**) and 2200 cm/s (**b**,**d**) in the occipital region.

**Figure 6:**
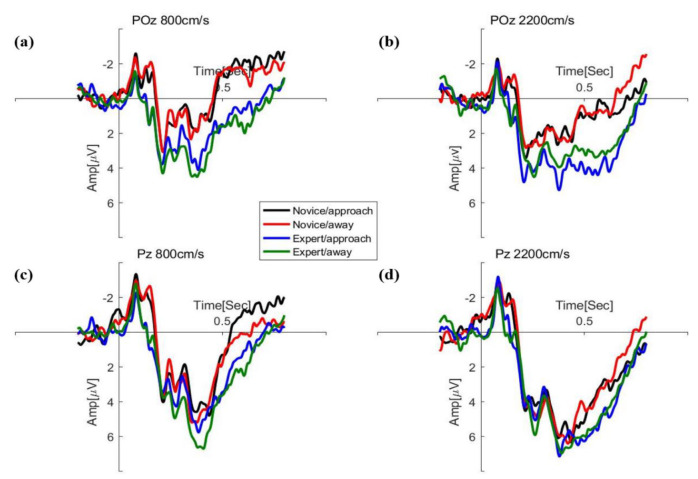
The grand–average waveform for expert and novice at speeds of 800 cm/s (**a**,**c**) and 2200 cm/s (**b**,**d**) in the parieto-occipital region.

ERP data on electrode sites selected for further statistical analysis included P5, P6, PO7, and PO8 for P1, N2, Oz, Pz, and POz for P2, and CPz, Cz, Pz, and FCz for P300. Peak amplitudes (measured baseline–peak) and corresponding latencies were detected in four windows following stimulus onset, including 80~140 ms for P1, 160~200 ms for N2, and 200~260 ms for P2 in Figure 4, and 280~550 ms for P300. Results of the repeated measures ANOVA analysis of amplitudes and latency in the ERP components are shown in Figure 5 and Figure 6.

*P1 amplitude analyses.* The current study results showed that the occipital–temporal region had a significant effect on direction, F (1, 26) = 6.98, *p* = 0.014, and the amplitude of “approach” (1.27 ± 0.35) was smaller than that of “away” (1.61 ± 0.38). The interaction between the movement direction and speed was significant at electrode points P5 and PO7, F (7,182) = 2.59, *p* = 0.014. Upon simple effect analysis, it was demonstrated that an increase in movement led to decreased amplitude when the sphere was approaching. Furthermore, significant differences were reported at 1000 cm/s, 1200 cm/s, and 1800 cm/s (*p* = 0.032). Mean amplitude of the novice group (2.34 ± 0.64) was significantly higher than that of the expert group (0.61 ± 0.64), F (1, 26) = 3.67, *p* = 0.056. However, the findings of the current study showed a significant interaction between the movement direction and the group, F (1, 26) = 11.16, *p* = 0.013. Results of the simple effects analysis showed that the amplitude of “approach” in the expert group (1.65 ± 0.44) was significantly smaller compared to that of “away” (2.21 ± 0.51) (*p* = 0.002).

*P1 latency analyses.* Primary ANOVA analysis of posterior P1 peak latency showed a significant interaction of movement direction and group, F (1, 26) = 4.57, *p* = 0.042. Simple effect analysis reported a significantly higher latency of “approach” in the expert group (110.60 ± 1.77) than that of “away” (108.16 ± 1.61) (*p* = 0.03). However, significant differences among the novice participants were not observed in the current study.

*N2 amplitude analyses.* There was a significant interaction between movement direction and speed in the occipital–temporal region, F (7, 182) = 5.12, *p* < 0.001. In addition, simple effect analysis showed significant differences in amplitude among different speeds when the ball was far away (*p* < 0.001). However, the difference was not significant when the ball was close. At a velocity of 2000 cm/s or 2200 cm/s, amplitude was significantly smaller when the sphere approached than when it moved away (*p* = 0.015).

*N2 latency analyses.* Findings of the current study indicated a marginally significant difference between latency period of approaching (185.59 ± 01.97) compared to that of away (182.88 ± 1.95). F (1, 26) = 3.90, *p* = 0.059.

*P2 amplitude analyses.* The significant main effects of speed, F (7, 182) = 3.84, *p* < 0.001, were observed at occipital sites. Notably, faster movements were associated with smaller amplitude. A significant interaction of movement direction and speed, F (4.57, 118.71) = 2.12, *p* < 0.001, was reported in the current study. Simple effect analysis showed significant differences in amplitude at different speeds (*p* < 0.001) when the sphere was far away. In addition, at a velocity of 2000 cm/s or 2200 cm/s, amplitude was significantly greater when the sphere approached than when it moved away (*p* < 0.001).

*P2 latency analyses.* The results of the current study showed significant differences among groups for the P2 latency, F (1, 26) = 5.65, *p* = 0.025. Novice latency (208.28 ± 1.55) was greater than expert latency (203.06 ± 1.5). The simple main effect of the movement direction was significant, F (1, 26) = 4.40, *p* = 0.046. The latency period of “approaching” (205.66 ± 1.72) was smaller than that of “away” (208.68 ± 1.08).

*P300 amplitude analyses.* The findings of the current study showed a significant main effect of movement direction at the parietal region, F (1, 26) = 14.55, *p* = 0.001. The approaching amplitude (5.47 ± 0.74) was smaller than the “away” amplitude (6.10 ± 0.75). Notably, the simple main effect of speed was significant, F (7, 182) = 5.55, *p* < 0.001, and was an indication that faster speeds correspond to greater amplitude.

*P300 latency analyses.* The significant main effect of speed, F (4.61, 119.77) = 3.08, *p* = 0.014, was reported at the parietal sites. Faster speeds correspondingly led to longer latency periods. Significant interaction of movement direction and speed, F (7, 182) = 2.10, *p* = 0.046, was noted in the current study. Simple effect analysis showed that the latency of different speeds was significantly different (*p* < 0.001). At speeds of 1600 cm/s, 1800 cm/s, 2000 cm/s, or 2200 cm/s, the latency period when the ball was approaching was significantly smaller compared to the latency period when the ball was far away (*p* = 0.005).

## 4. Discussion

We investigated the characteristics of motion-in-depth perception of tennis experts at different speeds of the bioelectric function. We postulated that participating in systematic long-term training in tennis will improve the perception of motion-in-depth. The findings of the study showed that experts process visual stimulus information faster compared to novices.

Although response time was faster, the accuracy decreased. Brenner and Smeets proposed that speed is the main determining factor used to determine when to intercept and hit the target [33]. Furthermore, for fast-moving objects, individuals act faster to compensate for delays in response. The decline in accuracy rate may be due to the fact that, at faster speeds, an athlete occupies a certain amount of attention resources to respond quickly. In addition, the speed–accuracy trade-off is reduced when the response time decreases [34]. The observation that faster speeds corresponded to larger P300 amplitude is consistent with the study by Ling Wang [26]. However, findings on latency were divergent. Faster speeds resulted in longer latency. At faster speeds of depth motion, more attention resources are occupied, and classification and recognition become more difficult than at low speeds. Thus, it takes longer to identify and evaluate stimuli. The effects of different changes in speed on human perception of motion-in-depth indicate that gradual increase in speed results in an acceleration of human perception of motion-in-depth. However, accuracy rate will decrease and EEG response will be significantly different, which is consistent with expectations of the first research hypothesis of the current study.

The activation degree of distant movement in the occipital–temporal area is significantly greater than that of near movement. This finding confirms that the occipital–temporal area is indeed related to primary visual information processing. Ordinary people feel that objects close to themselves are not the same as objects far away from them. The human visual system is more sensitive to the expansion of the convex circle, which produces an impression close to the object [32]. In the current study, the occipital–temporal regions P1 and N2 were reported to be significantly smaller in amplitude when approaching, and the latency period when approaching was longer than away. This is consistent with the conclusions of Ling Wang [26]. Our findings on the component analysis of P2 showed that faster movement of the sphere reduced the P2 amplitude. The effect of the speed factor is similar to the effect of the size change. Therefore, the findings of the current study postulate that the information processing of the speed factor during the depth motion process correlates with the information processing process of the size factor. Analysis of the late components in the occipital region indicated that an increase in speed necessitated the investment of more cognitive resources in the depth motion process to update the representation of working memory and slow the classification and evaluation of stimulation.

Significant differences in the response time of the expert group were not reported in this study, although significant differences in the novice group were reported. A P1 component data analysis demonstrated that the expert group activated an earlier and stronger response when the ball was away, whereas the novice group did not. Smith [35] found that the P1 component is mainly related to early attention distribution, and that it is more likely to be related to pattern processing. This implies that experts utilize more attention resources when the deep movement is far away, and that the response of the first-level visual information processing area is more sensitive. This means that experts can more quickly allocate attention resources to the process of observing the movement of the ball after hitting the ball. Nakamoto and Mori proposed that a reduction of a few milliseconds in visual processing time in tennis provides better accuracy in each shot, which is a significant advantage [36].

The P2 component analysis revealed that the occipital region has a sports expertise effect, which is consistent with findings of a study by Chenxi Jin [37]. The novice group had a longer latency than the expert group. The behavioural results of this study showed that a significant difference existed between the “approach” and “away” in the novice group’s reaction time, whereas the difference in the expert group was not significant. The novice group had a significantly longer reaction time when determining “approach” than when determining “away”, but the expert group had a non-significant difference in reaction time for both directions. When a stimulus is applied to the senses, the tennis expert can quickly recognize the stimulus features during the perceptual process and then encode and compare the information in the brain to make an accurate response. Most of the novices reported at the end of the experiment that they always panicked when facing a ball that approached them quickly and could not recognize and make judgments as calmly as the tennis experts. We have added the suggested content to the manuscript upon discussion.

The scanning and extracting information processing abilities of expert athletes in fast ball sports are superior to novices and have more accurate judgment. However, the visual information presented in the current study was relatively single. Some researchers reported that experts have faster optic nerve signal transmission than novices [38,39]. However, the visual pyramid theory [22] affirms that the completion of the current study may only require basic visual abilities and does not require high-level visual–motor abilities. This implies that it can also be done by novices with the same speed and accuracy. The findings of the current study revealed that experts often use strategies to ensure the correct rate. In addition, expert athletes pay attention to their accuracy rate and ignore the speed of their reaction, which may explain why the behavioural responses of expert athletes are not significantly different than that of novices. Our results of pairwise comparisons showed that the response time of expert athletes in each speed case was shorter than that of the novice, although not significantly.

The findings of the current study showed that athletes performed better in processing speed and in processing stimulus information. This is consistent with results from previous studies that showed better processing speed and selective attention [36,37,40]. The rapid response of tennis players should be related to the way the central nervous system allocates cognitive resources during processing. The present study makes several contributions to tennis training. Importantly, our findings demonstrated that experts have short latencies; even the lab tasks had nothing to do with their exercise environment.

## 5. Conclusions

The current study showed that the speed of sphere motion significantly affects the behavioural response of motion-in-depth perception. Faster ball movements lead to faster individual responses, which results in low accuracy. Tennis experts outperform novices in information processing speed, which may be related to the way the central nervous system allocates cognitive resources during processing. This cognitive ability is reflected in the relevant sports field or specific sports situations and can play a wider role in the general cognitive process.

## Data Availability

The data included in this research are available upon request.
